# Supplementary material for: Characteristics of the First Protein Tyrosine Phosphatase with Phytase Activity from a Soil Metagenome
Source: Genes (Basel). 2019 Jan 29;10(2):101. doi: 10.3390/genes10020101 (PMC6409689; doi:10.3390/genes10020101)
Supplement: Supplementary file 1 [file genes-10-00101-s001.pdf]

Table S1. Protein sequence similarities of deduced gene products encoded by the insert of pLP16

ORF number	No. of encoded amino acids	Accession no, closest similar protein, organism, (no. of encoded amino acids),	Percent identity to the closest similar protein (BlastP)
pho16B	376	WP_042381880.1,protein-tyrosine phosphatase *Streptacidiphilus melanogenes,* (372)	(79%)
Orf 02	325	WP_042417920.1, LacI family transcriptioN/Al regulator *Streptacidiphilus anmyonensis,* (325)	(82%)
Orf03	117	WP_018557579.1, hypothetical protein *Streptomyces sp*. BoleA5 (883)	(53%)
Orf04	119	RJW72075, vacuolar protein sorting-associated protein 53 -like protein *Clonorchis sinensis* ,(1358)	(29%)
Orf05	37	PZN39371.1, sulfoN/Ate ABC transporter ATP-binding protein, Proteobacteria bacterium, (246)	(63%)
Orf06	96	WP_099477000.1, TerC family protein *Paenibacillus ihbetae,* (221)	(34%)
Orf07	38	AAW27310.1, SJCHGC02811 protein *Schistosoma japonicum,* (133)	(45%)
Orf08	102	No BLAST hits found	N/A
Orf09	130	XP_016657880.1, uncharacterized protein LOC100159331 isoform X3 *Acyrthosiphon pisum,* (6853)	(42%)
Orf10	50	No BLAST hits found	N/A
Orf11	818	AWN00235.1, hypothetical protein, uncultured organism, (819)	(99%)
Orf12	35	WP_109505691.1, class I SAM-dependent methyltransferase *Nocardioides sp. YIM ART13,* (270)	(65%)
Orf13	200	No BLAST hits found	N/A
Orf14	41	No BLAST hits found	N/A
Orf15	109	No BLAST hits found	N/A
Orf16	102	No BLAST hits found	N/A
Orf17	63	OHB94183.1, transcription termination factor NusA *Planctomycetes* bacterium, (358)	(28%)
Orf18	191	XP_001794145.1, hypothetical protein SNOG_03588 *Parastagonospora nodorum SN15* (641)	(36%)
Orf19	118	PQE15591.1, Heavy metal tolerance protein *Rutstroemia sp. NJR-2017a BBW,* (1031)	(47%)
Orf20	25	KMP12518.1, hypothetical protein UZ36_00105 *Nitrospina sp*. SCGC, (342)	(100%)
Orf21	40	No BLAST hits found	N/A
Orf22	45	No BLAST hits found	N/A
Orf 23	410	WP_090595989.1, methyltransferase domain-containing protein *Auraticoccus monumenti,* (274)	(54%)
Orf24	140	No BLAST hits found	N/A
Orf25	82	No BLAST hits found	N/A
Orf26	133	No BLAST hits found	N/A
Orf27	48	No BLAST hits found	N/A
Orf 28	239	ONH24413.1, hypothetical protein BL253_30125 *Frankia asymbiotica,* (395)	(80%)
Orf29	64	WP_098051725.1, LacI family transcription regulator *Pantoea agglomerans,* (341)	(39%)
Orf30	105	No BLAST hits found	N/A
Orf31	117	No BLAST hits found	N/A
Orf32	74	No BLAST hits found	N/A
Orf33	49	No BLAST hits found	N/A
Orf34	69	No BLAST hits found	N/A
Orf35	63	No BLAST hits found	N/A
Orf36	110	KQB60501.1, hypothetical protein AE621_04680 *Acidovorax sp*, SD340 (145)	(42%)
Orf37	186	No BLAST hits found	N/A
Orf38	74	WP_125089351.1, hypothetical protein *Saccharopolyspora sp H219,* (134)	(52%)
Orf39	26	PYO22201.1, DNA-3-methyladenine glycosylase I , *Candidatus Rokubacteria,* 194	(100%)
Orf40	186	No BLAST hits found	N/A

N/A: not applicable

Supplementary Table S2. Rank of templates representing the top ten threading PDB templates used by I-TASSER.*

Rank	PDB Hit	Iden1	Iden2	Coverage	Norm. Z-score
1	2oz5	0.33	0.25	0.62	1.43
2	1ywf	0.32	0.23	0.60	3.46
3	5vgr	0.09	0.18	0.93	1.36
4	2oz5	0.33	0.25	0.62	1.87
5	4rkk	0.17	0.16	0.64	1.89
6	1ywf	0.33	0.23	0.60	4.96
7	1ywf	0.33	0.23	0.61	4.15
8	5gnr	0.10	0.17	0.87	1.34
9	2oz5	0.32	0.25	0.62	2.06
10	4rkk	0.14	0.16	0.83	1.12

*Iden1 is the percentage sequence identity of the templates in the threading aligned region with the query sequence. Iden2 is the percentage sequence identity of the whole template chains with query sequence. Coverage of the threading alignment is equal to the number of aligned residues divided by the length of query protein. Norm. Z-score is the normalized Z-score of the threading alignments. The top 10 alignments reported above (in order of their ranking) are from the following threading programs: 1: MUSTER, 2: FFAS-3D, 3: PROSPECT2, 4: SPARKS-X, 5: HHSEARCH, 6: HHSEARCH2, 7: HHSEARCH I, 8: PROSPECT2, 9: Neff-PPAS, 10: SP3.

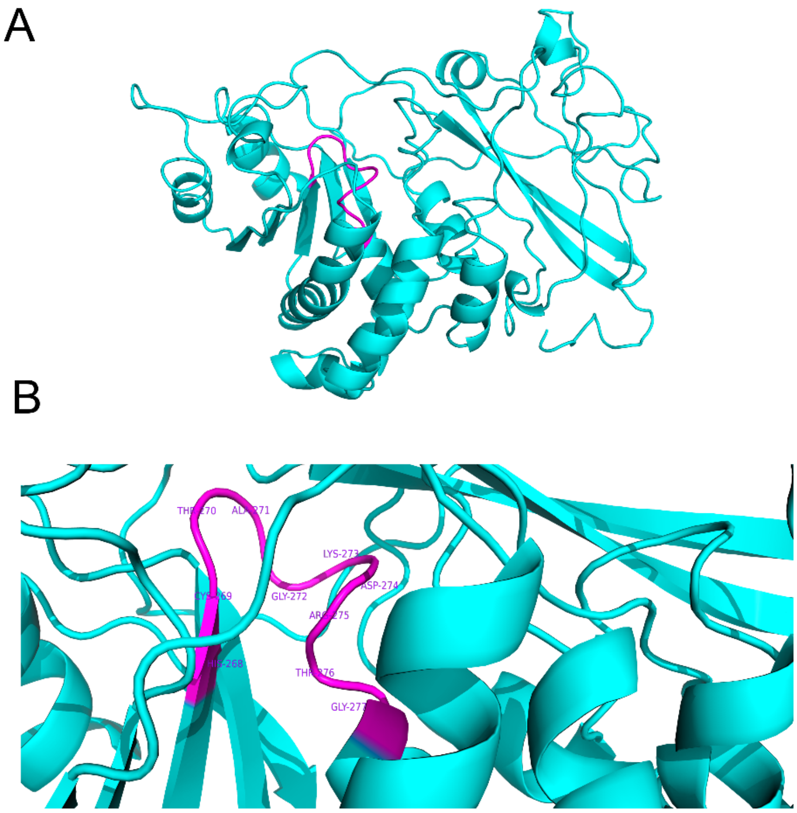

Supplementary Figure S1. A) Best 3D structure model of the Pho16B protein, predicted by I-TASSER and edited with Pymol 2.2, calculated C score -1.22. B) Amino acids involved in the MptpB-loop in Pho16B.
